# *LIVEtools* : an R Package for Visualizing *C. elegans* Embryo Cell Tracking Data

**DOI:** 10.17912/micropub.biology.001933

**Published:** 2026-03-25

**Authors:** Yuntian Gan, Sreekar Kutagulla, John Isaac Murray, Priya Sivaramakrishnan

**Affiliations:** 1 Department of Genetics, Perelman School of Medicine, University of Pennsylvania, Philadelphia, PA; 2 Center for Computational and Genomic Medicine, Children's Hospital of Philadelphia, Philadelphia, PA

## Abstract

Automated cell tracking in the *C. elegans* embryo using the StarryNite software pipeline has been a powerful tool to quantify the embryonic lineage, to define the spatiotemporal dynamics of gene expression, and to characterize developmental defects in mutants. The raw lineage traces are typically curated and visualized using the Java-based tool AceTree. However, these legacy visualization tools are often hard to deploy, are unstable on modern computer systems, and lack flexibility, especially for batch processing. *LIVEtools* is an R package that aims to simplify and automate *C. elegans* cell-tracking data visualization.

**Figure 1. Illustration and outputs of LIVEtools functionalities:** (A) Flowchart describing the main and optional steps to utilize *LIVEtools* code. 4D confocal images are processed through StarryNite and AceTree to obtain a data table with information on “cell”, “time”, “x”, “y” and “z” coordinates, and optionally gene expression, that serves as the starting point for *LIVEtools* -based analysis. Workflows exist to process a single embryo at a time or a group of embryos in batch as indicated. (B) Automated axis rotation. Top left: 3D visualization of a “raw” embryo dataset before rotation. Top right: the same embryo after rotation to align the AP axis with the x-axis and DV with the z-axis, using the lineages Cxp (posterior), Cxaa (dorsal), MSap (right) and MSpp (left) as axis landmarks. Bottom left (dorsal aspect) and right (anterior aspect) show 3D visualizations of post-rotated embryos with cell groups used for rotation colored as shown in the legend (cell colors are customizable). For DV rotation, left, right, and dorsal groups were specified, resulting in three candidate dorsal orientations based on each group (solid lines) which were averaged to guide the final rotation. (C) A rotated embryo 3D plot colored by mean reporter expression level. Selected lineages/cells can be highlighted and given custom marker shapes, with adjustments for color range (min/max) as needed. (D) Expression of a fluorescent reporter in the MS lineage (with user-defined root and end times) generated using the *plot_trees* function. (E) 3D positions of cells from the specified lineages in a wild-type (top) and *lit-1* RNAi-treated (bottom) embryo. (F) Expression level versus time for a *pha-4* expression reporter across many replicate embryos/conditions after batch processing and time alignment. Expression values are averaged across cells from arbitrary user-specified lineages (in this case ABalp, ABara, ABplpappp, ABprpappp and their descendants).

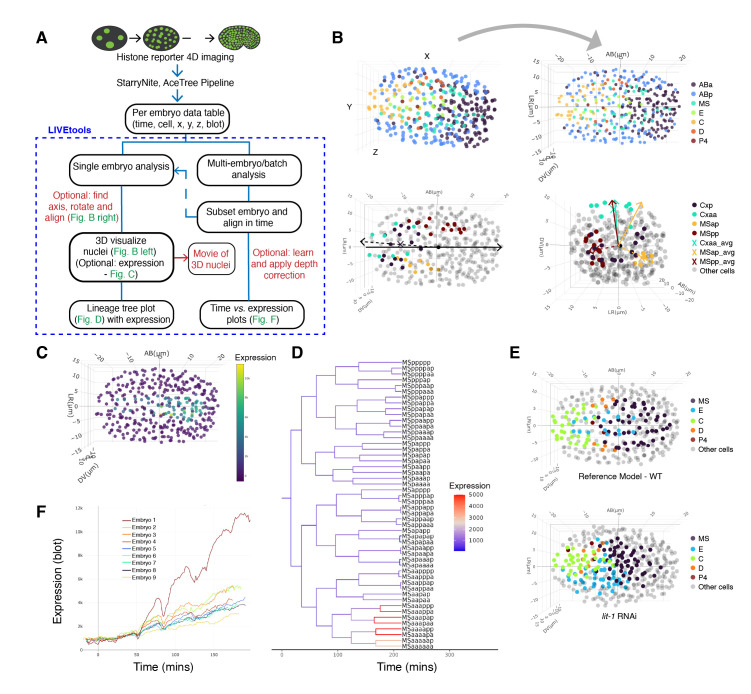



## Description

Embryos of the nematode *Caenorhabditis elegans* develop through a fully mapped invariant lineage (Sulston et al., 1983) . This lineage has proven to be a powerful experimental tool, and methods to automatically track the lineage from live imaging 4D microscopy data have allowed researchers to define the spatiotemporal dynamics of gene expression across the lineage (Araya et al., 2014; Ma et al., 2021; Mace et al., 2013; Murray et al., 2008, 2012; Sarov et al., 2012) . Applying these approaches to mutants or other perturbed embryos can identify defects in the lineage and in cellular gene expression or position (Du et al., 2015; Ho et al., 2015; Moore et al., 2013; Murray et al., 2022; Rumley et al., 2022; Walton et al., 2015) .

A common analysis pathway involves imaging embryos where all nuclei express a fluorescently tagged histone, and using the StarryNite analysis program for cell tracking (Bao et al., 2006) . The resulting cell lineage tracks can be further curated and visualized in the Java program AceTree (Boyle et al., 2006) . AceTree allows viewing expression, lineage, and cell positions, and fixing of any tracking errors made by StarryNite. AceTree also includes visualization tools, including exporting lineage trees as image files, displaying the 3D positions of cells as model projections, and plotting expression versus time along a lineage trajectory. However, these tools have limited flexibility and scalability due to their hard-coded nature and scale poorly due to the need to interact with a graphical user interface separately for each embryo. Furthermore, the original Java3D-based 3D viewer is incompatible with modern computers due to requirements for obsolete Java versions (Java 6) and libraries (Java3D). More recent updates to AceTree (Katzman et al., 2018) increased stability and replaced the obsolete 3D viewer with a new version based on WormGuides (Santella et al., 2015) , but reproducible figure design in AceTree is still inefficient.

Here, we present a codebase, *LIVEtools,* that allows efficient and reproducible manipulation and visualization of *C. elegans* lineage tracing data in the widely used R statistical programming language. This toolkit works from a simple tabular data format for each embryo, as typically produced by the StarryNite pipeline, and can also directly parse the raw StarryNite format (.zip) files. The user can incorporate additional metadata such as voxel size to convert between pixel and micron coordinates, and timestamp data to convert ordinal time points to elapsed time. A Sulston-compatible cell search tool allows visualization of highly customizable cell selections. Since images can be acquired in any orientation, *LIVEtools* can automate rotation of 3D views to a common orientation (e.g. dorsal aspect) and correct reduced expression intensity due to imaging depth.&nbsp;

The main features (illustrated in the Figure) are:

For batch processing, there are built-in functions to do the following (and custom batch processing is made possible using custom R scripts):

1. Simultaneously subset embryos.

2. Align the time between multiple embryos by developmental events (such as to compare expression after the birth of a particular blastomere).

3. Learn and correct for reporter signal loss over depth (z) due to imaging artifacts.

4. Plot reporter expression over time in the same trajectory between embryos to allow comparative expression analysis.

5. Save modified embryo data (rotated, depth-corrected).

*LIVEtools* enables early data inspection and efficient batch processing of arbitrary collections of embryo data. Use of common data processing will also simplify generation of reproducible analyses and figures for journal articles.

We illustrate these functions in (Fig 1. B-F). Fig. 1B illustrates 3D visualization of nuclei and the rotation function, using known or user-supplied cells to define relative positions to center the embryo. Defined orientations can be applied to all time points. Cells in the 3D plot can be colored by reporter expression ( Fig. 1C ), which can also be visualized through plotting of lineage trees ( Fig. 1D ). Average expression in specific lineages can be compared across conditions over time, after aligning embryos ( Fig. 1F ). 3D visualization can help interact with and identify position defects in mutant or perturbed embryos ( Fig. 1E ). The accompanying GitHub repository includes the code and examples to create these figures, as well as vignettes describing how to perform additional visualization.

## Methods

The methods are included and documented in detail in the GitHub repository and conceptually summarized here.

**File formats and loading**

The user can load data from a StarryNite-format file or a file in “CD” format. CD files are comma-separated text files, where the first line has column headers and additional lines have cell-time points. Required columns are “cell”, “time”, “x”, “y” and “z”. By default, expression-plotting functions will use a column called “blot,” but other quantitative columns with user-specified names can be selected as well. If a “TIME” file is provided, with columns including the ordinal time point and elapsed time, the true time values can be used for downstream plotting.

**3D rotation**

Embryo rotation is rigid-body, based on default or user-specified markers of the AP and DV axes such that the AP axis is aligned with the x-axis and DV with the z-axis. Briefly, at a specified time point, a transform matrix is defined that will align the AP axis (defined using PCA to identify the long axis of the embryo, with the specified cell groups ensuring anterior=positive x), and will rotate the embryo around this AP axis such that the specified dorsal and ventral cell group centroids are aligned with the z-axis (or optionally, left and right cell groups are aligned with the y-axis). Note that since the DV and LR axes are linked, providing markers for both axes will result in a solution that minimizes the total angular deviation of the provided cell groups from the y- and z-axis, likely meaning that neither will correspond perfectly to the corresponding axis. Finally, this transform matrix is applied either to the specified time point, or all time points to give a full-embryo rotation.

**Depth correction**

Depth correction of expression values relies on using replicate embryos (prior to 3D rotation to a common axis). *LIVEtools* learns an exponential decay model for the relationship of expression with z position (depth) across the replicate embryos provided, then uses this to correct expression of the specified embryos to a common reference depth, as previously described (Ma et al., 2021; Murray et al., 2008) . Once a depth correction model is learned, it can be applied to other embryos collected using equivalent imaging methods where the decay of signal with depth is similar to that learned on the training set.&nbsp;

**Plotting**

*LIVEtools* uses *ggtree* to output lineage trees as vector graphics, and *plotly* to output 3D visualizations. These 3D visualizations can be manually viewed and manipulated or automatically exported to images using provided functions based on the *reticulate* package and *kaleido* .

*LIVEtools* is available on GitHub at https://github.com/johnmurraylab/LIVE_tools as an R package. The code and example data required to generate the images in the figure are available on GitHub at: https://github.com/johnmurraylab/LIVEtools-paper .

## Data Availability

Description: v1.0 release of LiveTools. Resource Type: Software. DOI: https://doi.org/10.22002/zay8w-3dn76
